# Association between educational attainment and blood pressure in older adults: a study of two Finnish generational cohorts born 20 years apart

**DOI:** 10.1016/j.ijcrp.2025.200412

**Published:** 2025-04-22

**Authors:** Adriana Lääti, Oskari Somerpalo, Konsta Teppo, Jenni Vire, Matti Viitanen, Ville Langén

**Affiliations:** a Division of Medicine, Turku University Hospital and University of Turku, Turku, Finland, Kiinamyllynkatu 4-8, Turku, 20520, Finland; b Department of Geriatric Medicine, Turku University Hospital and University of Turku, Turku, Finland, Kunnallissairaalantie 20, Turku, 20700, Finland; c Faculty of Medicine, University of Turku, Turku, Finland, Kiinamyllynkatu 10, Turku, 20520, Finland; d Heart Centre, Turku University Hospital and University of Turku, Turku, Finland, Hämeentie 11, Turku, 20540, Finland; e Department of Neurobiology, Care sciences and Society, Karolinska Institutet, Huddinge, Sweden, Alfred Nobels Allé 8, Huddinge, 141 52, Sweden; f Theme Inflammation and Aging, Karolinska University Hospital, Sweden, Alfred Nobels Allé 8, Huddinge, Stockholm, 141 52, Sweden

**Keywords:** Aging, Blood pressure, Cohort study, Education, Hypertension, Socioeconomic disparities

## Abstract

**Background:**

This study compares the association between educational attainment and blood pressure (BP) in two Finnish cohorts of older adults, born 20 years apart.

**Methods:**

All 70-year-old residents of Turku, Finland, were surveyed in 1990 (1920-born TUVA cohort) and in 2010 (1940-born UTUVA cohort). Associations between education and BP were assessed using first ANOVA and post-hoc Tukey tests and then multiple linear regression, adjusted for age, gender, smoking, and body mass index. Analyses included 668 TUVA and 862 UTUVA participants.

**Results:**

In the TUVA cohort (67.7 % women, mean age 70.9), 77.7 % had primary education only, compared to 54.1 % in the UTUVA cohort (59.6 % women, mean age 71.4). ANOVA revealed a significant association between education level and diastolic BP in the UTUVA cohort (p = 0.04). All other ANOVA results were non-significant (p ≥ 0.14). Tertiary education did not have a significant association with BP (p ≥ 0.0544). In regression analyses, each additional year of education in UTUVA correlated with a 0.36 mmHg decrease in systolic BP (p = 0.01) and a 0.32 mmHg decrease in diastolic BP (p < 0.001).

**Conclusions:**

The 1920-born cohort demonstrated no significant differences in BP across education levels, whereas in the cohort born in 1940, higher education was associated with significant but small reductions in BP. These findings suggest that education may be linked to BP, but the absolute differences across education levels are modest. The relationship between education and BP is complex, influenced by lifestyle choices and healthcare access, and requires further exploration.

## Highlights

• Several risk factors for hypertension have been identified in older adults.
• However, the role of education in hypertension risk remains unclear in older adults.
• In a 1920-born cohort, education level showed no link to blood pressure.
• In a 1940 cohort, higher education was linked to slightly lower blood pressure.
• Tertiary education had no significant association with blood pressure.

## Graphical abstract

**Figure:**
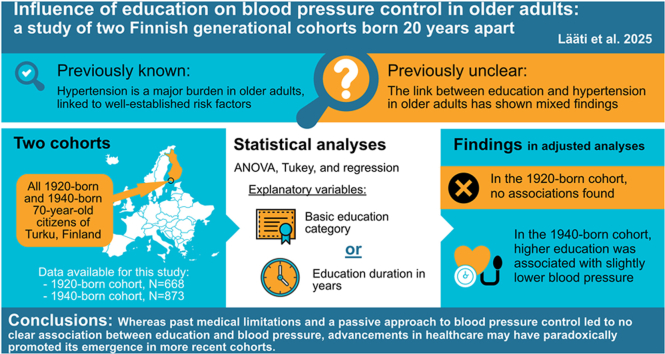

## 1 Introduction

Hypertension impacts over a billion individuals worldwide [1] and is widely acknowledged as the leading cause of the global disease burden [2]. The HYVET study, a landmark trial, demonstrated that antihypertensive treatment is clearly beneficial for adults over 80 years old [3].

The prevalence of hypertension correlates positively with age, particularly in those aged 65 years and older [4]. Beyond age, the development of hypertension is heavily influenced by several well-known risk factors, such as obesity, tobacco use, high salt intake, and sedentary lifestyle [5]. However, the specific impact of socioeconomic factors, such as educational level, on blood pressure (BP) control in older adults remains less clear.

Previous research in this domain is notably fragmented and limited, particularly in older patient cohorts. Regidor et al. identified a higher incidence of hypertension among older men with lower educational levels, though the causal factors remain unclear [6]. Meanwhile, Wagner et al. reported that hypertension was more prevalent in areas with lower educational levels in Brazilian older adults [7]. In contrast, Eshkoor et al. noted that educational level and marital status did not associate with the prevalence of hypertension in Malaysian older adults [8].

In this study, we aimed to enhance understanding of the relationship between education and BP by analyzing data from two population-based older adult generational cohorts born two decades apart, with data encompassing both pre- and post-HYVET eras. Specifically, we assessed the hypothesis that higher educational attainment has an association with lower BP persisting into older age.

## 2 Materials and methods

### 2.1 Participants

The study sample included individuals from two distinct birth cohorts: those born in 1920 and 1940. In 1990, the Turku Older Adults Study (TUVA) invited all 1920-born residents of Turku, in Southwest Finland, to participate in a prospective cohort study (n = 1503). After excluding refusals, institutionalized individuals, and those with incomplete questionnaires, 1032 (68.7 %) participants aged 70–71 were included in the initial study conducted between 1991 and 1992.20 years later, the second birth cohort, born in 1940, was invited to participate in the New Turku Older Adults Study (UTUVA), conducted in 2011 and 2012. A total of 1344 70-year-olds met similar exclusion criteria as in TUVA, resulting in 956 (71.1 %) participants in the UTUVA study. Finally, we excluded those without data for both systolic and diastolic BP readings, age, gender, smoking, body mass index (BMI), and basic school education level at baseline, resulting in 668 TUVA participants and 862 UTUVA participants for the analyses of the present paper.

### 2.2 Health interview and health examination

Both cohorts underwent a similar examination protocol, which included postal questionnaires, interviews, and clinical examinations and has been described in detail in prior publications [[9], [10], [11]]. In the baseline TUVA study, health examinations were conducted at health centres by experienced general practitioners and research nurses. In the baseline UTUVA study, examinations were carried out at the research centre, where BP measurements were taken by research physicians.

### 2.3 BP measurements

As the baseline study of UTUVA did not have consecutive sitting BP measurements available, we opted to use only the first BP reading for systolic and diastolic BP for both TUVA and UTUVA. The first reading was taken as sitting BP at the baseline TUVA study and as supine BP in the UTUVA study. BP was measured using the auscultatory method with a mercury sphygmomanometer at the baseline TUVA study. For the baseline UTUVA study, an oscillometric OMRON device (Omron Matsusaka Co., Kyoto, Japan) was used. Hypertension was defined as either as having systolic BP ≥ 140 mmHg and/or diastolic BP ≥ 90 mmHg, self-reported hypertension, or confirmed use of antihypertensive medication without indications other than hypertension. Other indications comprised heart failure, coronary artery disease, previous acute myocardial infarction, arrhythmia (defined as atrial fibrillation in the UTUVA cohort), other cardiac diseases, and kidney disease.

### 2.4 Education variables

The data on education levels were collected as a categorical variable through a questionnaire, with participants indicating their highest level of basic education. The original categories were "Less than primary education," "Primary education," "General lower secondary education," and "General upper secondary education." Due to the small sample size in the "Less than primary education" category (only 9 participants in TUVA and 3 in UTUVA), this category was merged with "Primary education" to form a combined category named "Primary education or less."

Basic education duration was initially recorded as a continuous variable through a questionnaire. Invalid entries were detected based on mismatches between education level and reported duration compared to the expected durations for each education level in Finland. Missing data for duration were present in 274 cases (41 %) in TUVA and 164 cases (19 %) in UTUVA. Invalid durations were identified in 89 cases (13 %) for TUVA and 86 cases (10 %) for UTUVA. Mean durations for each education level were calculated using the valid data available within each cohort. Specifically, these mean durations were 3 years for "Less than primary education," 7 years for "Primary education," 10 years for "General lower secondary education," and 12 years for "General upper secondary education." These calculated means aligned with the expected durations for each education category in Finland and were used to impute missing or invalid values. The final education duration variable combined the basic education duration and any reported vocational education duration. Additionally, a separate education duration variable without imputations was calculated for sensitivity analyses.

In addition, a categorical variable for having completed tertiary education was available exclusively for the 1940-born UTUVA cohort but not for the 1920-born TUVA cohort.

### 2.5 Antihypertensive medications

In brief, antihypertensive medications were identified through questionnaires, individual interviews, and prescriptions, and classified using the ATC codes. All diuretics, excluding loop diuretics, were considered antihypertensive medications. A detailed description of the classification process can be found in our earlier paper [12].

### 2.6 Statistical analyses

Statistical analyses were performed using R, version 4.3.0 (R Core Team, Vienna, Austria). In the main analyses of this paper, ANOVA was conducted within each cohort to compare BP readings across different education categories, followed by post-hoc Tukey tests to identify specific group differences. Regression analyses were used to examine the relationship between education duration (as a continuous variable) and BP within each cohort, starting with crude models and subsequently adjusting for age, gender, smoking, and BMI. Additionally, linear regression models incorporating restricted cubic splines with three knots at the 25th, 50th, and 75th percentiles were fitted to generate regression plots visualizing the relationship between education duration and BP. Unpaired t-tests and chi-square tests were applied to compare covariates between the TUVA and UTUVA cohorts. A p-value of less than 0.05 was considered statistically significant for all analyses.

### 2.7 Ethics

The study protocol for the TUVA study was approved by the City of Turku Ethics Committee on Health Care on December 19, 1990 (Record No. 2/90). The UTUVA study received similar approval from the Ethical Committee of the Hospital District of Southwest Finland on February 16, 2010 (ETMK: 2/180/2010). All participants provided informed consent in accordance with the Declaration of Helsinki.

## 3 Results

**Fig. 1:**
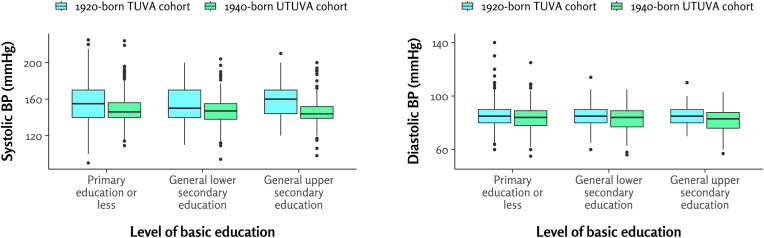
Distribution of systolic (left) and diastolic (right) blood pressure by level of basic education in older adults. BP, blood pressure.

The characteristics of both study cohorts are presented in Table 1. The prevalence of hypertension was 87.6 % in the 1920-born TUVA cohort and 84.2 % in the 1940-born UTUVA cohort. 33.5 % of participants in the TUVA cohort were on antihypertensive treatment, compared to 55.9 % in the UTUVA cohort. A more detailed comparison of BP-related variables between these cohorts is provided in our earlier publication [12]. The majority of participants in both cohorts were women, comprising 67.6 % of the 1920-born TUVA cohort and 59.6 % of the 1940-born UTUVA cohort. The mean age of participants was 70.9 years in the TUVA cohort and 71.4 years in the UTUVA cohort. In both cohorts, most participants had completed only primary education or less, with 77.7 % in the TUVA cohort and 54.1 % in the UTUVA cohort. Conversely, only 7.8 % of TUVA participants and 22.5 % of UTUVA participants reported having attained general upper secondary education. Fig. 1 presents boxplots of systolic and diastolic BP across the basic education groups.

**Table 1:** Characteristics of the study cohorts.

Variable	1920-born TUVA cohort	1940-born UTUVA cohort	p
n	668	862	
Age (years)	70.9 ± 0.4	71.4 ± 0.2	<0.001
Women	452 (67.7 %)	514 (59.6 %)	0.001
Basic education			<0.001
Primary education or less	519 (77.7 %)	466 (54.1 %)	
General lower secondary education	97 (14.5 %)	202 (23.4 %)	
General upper secondary education	52 (7.8 %)	194 (22.5 %)	
Education duration (years)	8.3 ± 2.8	10.9 ± 4.1	<0.001
Tertiary education	no data	98 (11.4 %)	
Systolic BP (mmHg)	156 ± 21	148 ± 16	<0.001
Diastolic BP (mmHg)	86 ± 10	83 ± 8	<0.001
Hypertension	585 (87.6 %)	726 (84.2 %)	0.08
Use of any antihypertensive medication	224 (33.5 %)	482 (55.9 %)	<0.001
Body mass index (kg/m2)	26.6 ± 4.0	27.5 ± 4.5	<0.001
Smoking status			0.37
Never smoked	398 (59.6 %)	484 (56.1 %)	
Quit	208 (31.1 %)	286 (33.2 %)	
Currently smoking	62 (9.3 %)	92 (10.7 %)	

Values are means ± standard deviations for continuous data and numbers and percentages for categorical data. BP, blood pressure. Education duration was defined as the total number of years of formal education, including both basic and vocational education.

### 3.1 ANOVA analysis between the level of basic education and BP

In the TUVA cohort, there was no significant difference in systolic (p = 0.44) or diastolic BP (p = 0.52) across education levels. Conversely, in the UTUVA cohort, while systolic BP showed no significant difference (p = 0.14), diastolic BP differed significantly across education levels (p = 0.04) (Table 2).

**Table 2:** Blood pressure, hypertension prevalence, and antihypertensive medication use by education level.

Cohort	Basic education or less	General lower secondary education	General upper secondary education	p-value
**1920-born TUVA cohort**				
Group size (n)	519	97	52	
Systolic blood pressure (mmHg)	156 ± 21	154 ± 18	159 ± 21	0.44
Diastolic blood pressure (mmHg)	86 ± 10	85 ± 10	85 ± 9	0.52
Prevalent hypertension	450 (86.7 %)	88 (90.7 %)	47 (90.4 %)	0.44
Use of any antihypertensive medication	169 (32.6 %)	36 (37.1 %)	19 (36.5 %)	0.61
**1940-born UTUVA cohort**				
Group size (n)	466	202	194	
Systolic blood pressure (mmHg)	149 ± 16	147 ± 16	146 ± 16	0.14
Diastolic blood pressure (mmHg)	84 ± 9	83 ± 9	82 ± 8	0.04
Prevalent hypertension	399 (85.6 %)	168 (83.2 %)	159 (82.0 %)	0.45
Use of any antihypertensive medication	267 (57.3 %)	108 (53.3 %)	107 (55.2 %)	0.64

Values are means ± standard deviations for continuous data and numbers and percentages for categorical data. Group comparisons used ANOVA for continuous and chi-square for categorical variables.

### 3.2 Post-hoc Tukey HSD test analysis for ANOVA test of differences in BP between basic education groups

In the TUVA cohort, there were no significant differences in systolic or diastolic BP across education levels (adjusted p for all ≥0.42). However, in the UTUVA cohort, diastolic BP was 1.79 mmHg lower in participants with general upper secondary education compared to those with primary education or less (adjusted p-value = 0.03). Systolic BP in the UTUVA cohort showed no significant differences across education levels (adjusted p ≥ 0.15 for all comparisons).

### 3.3 Regression analyses of the association between combined duration of basic and vocational education in years and BP

**Fig. 2:**
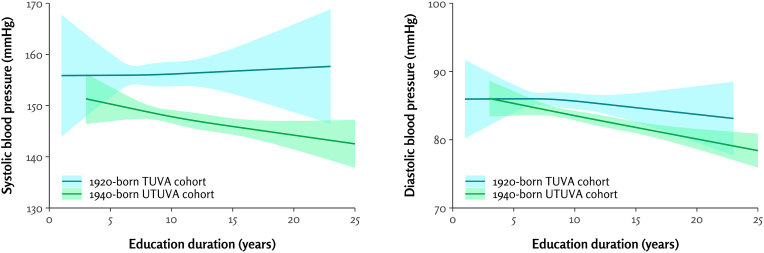
Association between educational level and systolic (left) and diastolic (right) blood pressure in the 1920-born and 1940-born older adult cohorts. Restricted cubic spline regression lines (solid lines) with 95 % confidence intervals (shaded areas) were used to model these associations. Education duration was defined as the total number of years of formal education, including both basic and vocational education.

In the 1920-born TUVA cohort, the adjusted associations between years of education and systolic (β = 0.31, p = 0.29) and diastolic BP (β = −0.04, p = 0.78) were not statistically significant (Table 3, Fig. 2).

**Table 3:** Regression analyses of the association of combined basic and vocational education duration (years) and blood pressure (mmHg).

Outcome variable	1920-born TUVA cohort			1940-born UTUVA cohort		
	β	SE	p	β	SE	p
Systolic blood pressure	0.31	0.29	0.29	−0.36	0.13	0.01
Diastolic blood pressure	−0.04	0.14	0.78	−0.32	0.07	<0.001

All models adjusted for age, gender, smoking, and body mass index. Education duration was defined as the total number of years of formal education, including both basic and vocational education.

In the 1940-born UTUVA cohort, adjusted analyses showed that each additional year of education was associated with a 0.36 mmHg lower systolic BP (p = 0.01) and a 0.32 mmHg lower diastolic BP (p < 0.001) (Table 3, Fig. 2).

As a sensitivity analysis, these regression models were rerun using an education duration variable without imputations; however, the results remained materially unchanged (data not shown).

### 3.4 BP differences by tertiary education status in the 1940-born UTUVA cohort

As data on tertiary education were not available for the 1920-born TUVA cohort, these analyses were conducted exclusively for the 1940-born UTUVA cohort. In this cohort, 11.4 % of participants had completed tertiary education, while the remaining 88.6 % had no tertiary education.

**Fig. 3:**
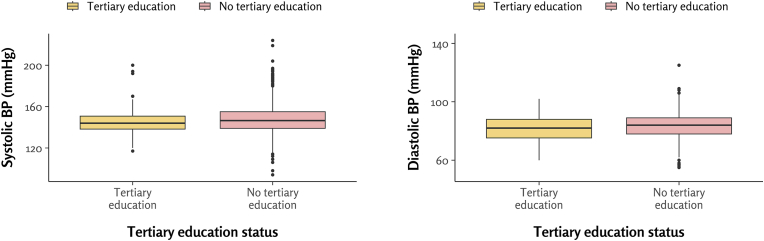
Distribution of systolic (left) and diastolic (right) blood pressure by tertiary education status in 1940-born older adults. BP, blood pressure.

The mean systolic BP (± standard deviation) was 145 ± 15 mmHg in participants with tertiary education, compared to 148 ± 16 mmHg in those without tertiary education (p = 0.12). Similarly, the mean diastolic BP was 82 ± 8 mmHg in the tertiary-educated group, while the non-tertiary group had a mean of 83 ± 8 mmHg (p = 0.0544) (Fig. 3).

### 3.5 Hypertension prevalence and antihypertensive medication use by basic education groups

No significant differences in hypertension prevalence or antihypertensive medication use were observed across education groups in either the 1920-born TUVA cohort (hypertension: p = 0.44, medication use: p = 0.61) or the 1940-born UTUVA cohort (hypertension: p = 0.45, medication use: p = 0.64) (Table 2).

## 4 Discussion

This population-based study aimed to evaluate whether BP control differs across education levels among the older population. We investigated this research question in two generational cohorts, each invited to join the study at the age of 70 years. We found that in the 1920-born TUVA cohort, both systolic and diastolic BP showed only minimal, statistically insignificant variation across different basic education levels. In the 1940-born UTUVA cohort, a similar pattern was observed for systolic BP; however, diastolic BP was significantly lower by a mean difference of 1.79 mmHg in individuals with upper secondary education compared to those with primary education or less. Additionally, regression analyses in the UTUVA cohort treating education as a continuous variable showed that each additional year of education was associated with a 0.36 mmHg reduction in systolic BP and a 0.32 mmHg reduction in diastolic BP. In our analysis of the 1940-born UTUVA cohort, which had data on tertiary education status, we found no significant differences in systolic or diastolic BP between those with and without tertiary education.

The relatively few earlier studies that have examined the link between education and BP in older adults have presented somewhat conflicting results. Wang et al. reported a strong inverse association between education and hypertension in the Chinese population, where more years of education were linked to a reduced risk of hypertension [13]. Similarly, Park et al. observed that individuals with lower educational attainment had a higher prevalence of hypertension compared to those with higher education [14]. Leng et al. also found that lower socioeconomic status, particularly education level, was associated with elevated BP [15]. In contrast, Kirschbaum et al. noted that the prevalence of hypertension was generally similar across educational groups in most countries, except in Southeast Asia, where higher education levels were positively associated with hypertension [16].

Differences between our findings and those of other studies may stem from variations in healthcare systems, accessibility, quality, and public health initiatives across countries. Finland's universal healthcare system, ensuring equitable access to services, likely contributes to more consistent health outcomes across different educational groups. The relationship between education and BP may also involve factors beyond access to medical care, such as stress levels and lifestyle habits. A lower level of education is often associated with higher stress and chronic stress [[17], [18], [19]], which is known to contribute to increased diastolic BP due to prolonged sympathetic nervous system activation [20,21]. Lower socioeconomic status has also been linked to unhealthy lifestyle habits [22], which may adversely affect BP. However, a higher level of education can lead to a sedentary job and lifestyle [23], which may adversely affect BP control [24]. Conversely, jobs that involve moderate physical activity could contribute to better cardiovascular health [23], assuming other lifestyle habits are not unhealthy. These factors, which may exert opposing influences, could affect the outcomes of studies examining the relationship between education and BP.

Examining generational cohorts provides insight into how education might influence BP, both in terms of its long-term effects on hypertension pathogenesis and factors affecting BP control at the time of assessment. In the 1920-born TUVA cohort, there was negligible, statistically insignificant variation in BP across education levels, likely reflecting limited medical advancements and a relatively passive approach to BP control in 1990. In contrast, the 1940-born UTUVA cohort showed significant, albeit minor, differences in BP across education levels, likely due to improved healthcare access and health knowledge by 2010 – changes that could, ironically, pave the way for emerging socioeconomic disparities.

This study has several limitations that should be considered when interpreting the results. First, this study cannot infer causality between education and BP due to its observational nature. Second, the findings were based on a Finnish population with universal healthcare, which may limit generalizability to countries with more stratified healthcare access. Additionally, the relatively small sample size and the use of data from a single urban area in Finland may further restrict the applicability of the results to other regions or more diverse populations. Relatedly, while the statistical power in the analyses based on education categories may be limited, the power with the continuous variable on education years is likely more sufficient. Moreover, survivor bias could affect our results, and the association between education and BP may differ within the same birth cohort if measured at an earlier age. We also focused primarily on education, without considering other socioeconomic factors such as income or occupation, which could offer a fuller understanding of BP disparities. Missing and imputed data may affect the reliability of the observed association between education duration and BP. However, analyses that utilized a categorical educational attainment variable were not subject to this limitation to a similar extent. Additionally, our sensitivity analyses, which repeated the regression models using an education duration variable without imputations, showed materially unchanged results. Another limitation is the difference in BP measurement methods between the two cohorts, with supine measurements in UTUVA and sitting measurements in TUVA. Previous research has shown mixed results on how these methods compare [25,26], and while this limitation must be acknowledged, direct comparison of BP values was not the focus of our study. The primary aim was to examine the association between education and BP, which we believe remains robust despite these measurement differences. Ideally, 24-h ambulatory BP monitoring would have provided a more comprehensive method for assessing BP in both cohorts, but it is more challenging to implement in large-scale surveys and was not available in this study. Sample homogeneity may have influenced the results. For instance, in the first sample, educational levels were predominantly uniform, with most individuals having only primary education. Furthermore, the minimal variability in educational duration, reflected in small standard deviations when analyzed as a continuous variable, likely restricted the ability to detect significant associations. For that reason, we consider it important that both categorical and continuous exposure variables were used to investigate the research question, as this approach helps provide a more comprehensive analysis despite the homogeneity of the sample. On the other hand, the potential for type I error should be considered due to these different types of analyses that were conducted to address the research question. Lastly, we did not account for certain potential confounders such as diet, physical activity, and genetic factors, which could influence the observed associations.

In conclusion, our study found a slight variation in the association between education and BP across two generational cohorts. In the 1920-born cohort, differences in BP were insignificant, while in the 1940-born cohort, higher education was associated with lower diastolic BP, and both systolic and diastolic BP decreased modestly with each additional year of education. The generational differences suggest that improved healthcare and knowledge could introduce modest BP differences. Overall, our study suggests that while education is linked to small BP differences in older adults, Finland's universal healthcare system helps mitigate the disparities seen in other countries. Further research is needed to explore these dynamics in diverse populations and healthcare systems.

## CRediT authorship contribution statement

**Adriana Lääti:** Writing – review & editing, Writing – original draft, Visualization, Methodology, Funding acquisition, Formal analysis, Conceptualization. **Oskari Somerpalo:** Writing – review & editing, Writing – original draft, Visualization, Methodology, Formal analysis, Conceptualization. **Konsta Teppo:** Writing – review & editing, Writing – original draft, Visualization, Supervision, Methodology, Funding acquisition, Formal analysis, Conceptualization. **Jenni Vire:** Writing – review & editing, Writing – original draft, Resources, Project administration, Methodology, Investigation, Funding acquisition, Data curation, Conceptualization. **Matti Viitanen:** Writing – review & editing, Writing – original draft, Supervision, Resources, Project administration, Methodology, Investigation, Funding acquisition, Conceptualization. **Ville Langén:** Writing – review & editing, Writing – original draft, Visualization, Supervision, Software, Project administration, Methodology, Funding acquisition, Formal analysis, Data curation, Conceptualization.

## Disclosure statement

VL: Speaker: Boehringer-Ingelheim. Other authors report no conflict of interest.

## Data availability statement

The analysis codes for this study have been deposited in the Zenodo repository with the following DOI 10.5281/zenodo.14197215.

## Funding

Dr. Viitanen was supported by The King Gustaf V's and Queen Victoria's Freemasons' Foundation, Perklén Foundation, and the State Research Funding of the wellbeing services county of Southwest Finland.

Dr. Teppo has received research grants from The 10.13039/100008723Finnish Medical Foundation and The Finnish Foundation for Alcohol studies.

Dr. Langén was supported by a grant from the State Research Funding of the wellbeing services county of Southwest Finland.

Dr. Lääti was supported by a grant from the Kunnanlääkäri 10.13039/501100016030Uulo Arhio Foundation, 10.13039/100018167Urmas Pekkala Foundation, and Betania Foundation.
